# Analytical validation of NeXT Personal^®^, an ultra-sensitive personalized circulating tumor DNA assay

**DOI:** 10.18632/oncotarget.28565

**Published:** 2024-03-14

**Authors:** Josette Northcott, Gabor Bartha, Jason Harris, Conan Li, Fabio C.P. Navarro, Rachel Marty Pyke, Manqing Hong, Qi Zhang, Shuyuan Ma, Tina X. Chen, Janet Lai, Nitin Udar, Juan-Sebastian Saldivar, Erin Ayash, Joshua Anderson, Jiang Li, Tiange Cui, Tu Le, Ruthie Chow, Randy Jerel Velasco, Chris Mallo, Rose Santiago, Robert C. Bruce, Laurie J. Goodman, Yi Chen, Dan Norton, Richard O. Chen, John M. Lyle

**Affiliations:** ^1^Personalis, Inc., Fremont, CA 94555, USA; ^*^Co-last authors

**Keywords:** circulating tumor DNA, whole genome sequencing, molecular residual disease, tumor-informed assay, analytical validation

## Abstract

We describe the analytical validation of NeXT Personal^®^, an ultra-sensitive, tumor-informed circulating tumor DNA (ctDNA) assay for detecting residual disease, monitoring therapy response, and detecting recurrence in patients diagnosed with solid tumor cancers. NeXT Personal uses whole genome sequencing of tumor and matched normal samples combined with advanced analytics to accurately identify up to ~1,800 somatic variants specific to the patient’s tumor. A personalized panel is created, targeting these variants and then used to sequence cell-free DNA extracted from patient plasma samples for ultra-sensitive detection of ctDNA.

The NeXT Personal analytical validation is based on panels designed from tumor and matched normal samples from two cell lines, and from 123 patients across nine cancer types. Analytical measurements demonstrated a detection threshold of 1.67 parts per million (PPM) with a limit of detection at 95% (LOD_95_) of 3.45 PPM. NeXT Personal showed linearity over a range of 0.8 to 300,000 PPM (Pearson correlation coefficient = 0.9998). Precision varied from a coefficient of variation of 12.8% to 3.6% over a range of 25 to 25,000 PPM. The assay targets 99.9% specificity, with this validation study measuring 100% specificity and *in silico* methods giving us a confidence interval of 99.92 to 100%.

In summary, this study demonstrates NeXT Personal as an ultra-sensitive, highly quantitative and robust ctDNA assay that can be used to detect residual disease, monitor treatment response, and detect recurrence in patients.

## INTRODUCTION

Tumor DNA shed into the patient’s bloodstream can be detected as circulating tumor DNA (ctDNA). When the patient undergoes surgical resection or other treatment, ctDNA is a powerful noninvasive tool for detecting molecular residual disease (MRD), monitoring for recurrence, or tracking therapy response. For example, post-surgery, the presence of ctDNA has been shown to predict disease recurrence [1–4] and lower overall survival in a wide range of cancers [5–15]. Detection of ctDNA raises the possibility of escalating treatment for patients earlier, with the goal of improving outcomes. For example, the CIRCULATE Japan study on resectable colorectal cancer stage II-IV, looked at using ctDNA status to determine whether to escalate or de-escalate treatment post-operatively [16]. The absence of tumor post-surgery, as indicated by the absence of ctDNA, potentially allows for the de-escalation of adjuvant treatment, thereby avoiding unnecessary toxicity to the patient, as well as saving on medication costs [4, 17–20]. In the DYNAMIC trial, patients negative for ctDNA were spared adjuvant chemotherapy. The cohort containing these patients had non-inferior 2-year recurrence-free survival compared to patients who received standard adjuvant chemotherapy, suggesting that ctDNA-negative patients could forgo chemotherapy without deleterious effect [19].

Several ctDNA assays have been shown to detect tumor recurrence earlier than radiological imaging owing to their sensitivity [3, 7, 21–25]. Such ctDNA assays have also predicted metastases in seemingly non-recurrent patients. In HR+ breast cancer patients who were serially tested, ctDNA was detected before distant metastasis was observed [26]. Because of their specificity and sensitivity in detecting recurrent disease, ctDNA assays are also useful in clinical trial design and recruitment [22, 27]. In addition, ctDNA assays help identify patients at higher risk of recurrence, such that treatment efficacy would be enhanced, thereby reducing sample size, time to trial completion, and cost for the study. The exclusion of low ctDNA patients who are not at risk of disease progression spares those patients from exposure to inappropriate therapy. During a clinical study, ctDNA can also be an early signal of drug efficacy, though further extensive clinical validation will be required before ctDNA assays can be used as a surrogate endpoint [28].

Changes in ctDNA levels following therapy can be indicative of disease response. After chemotherapy or radiological treatment of advanced cancers, a decrease in ctDNA was correlated with better patient response in a range of cancers [20, 29–33]. Conversely, increased ctDNA was associated with shorter progression-free survival [34].

In recent years, two major approaches have emerged for detecting ctDNA in cancer patients: tumor-informed and tumor agnostic approaches. Tumor-informed approaches utilize the patient’s tumor to create a custom panel specific for detecting ctDNA based on variants found in the tumor. Tumor agnostic approaches use a fixed panel assay design for all patients, and while convenient, are generally less sensitive compared to tumor-informed assays [35]. For these current ctDNA assays, limits of detection (LODs) typically range from 0.008 to 0.25% (or 80 to 2,500 in units of parts per million, PPM) [9, 36–39]. The lower the LOD of the assay, the more sensitive it is, and the earlier a residual or recurrent tumor could be detected. For example, it has been claimed that an LOD of 0.01% (100 PPM) or lower is required to detect metastatic recurrence in triple negative breast cancer [40]. Conversely, a ctDNA assay with less sensitivity may result in undetected tumor and consequently missed treatment opportunities [41, 42]. For example, in a recent study on resected CRC patients, one ctDNA assay detected tumor recurrence 53.3% of the time, no better than imaging and carcinoembryonic antigen (CEA), which had a 60.0% detection rate. Poor sensitivity of the assay thus led the authors to conclude that the ctDNA assay may not be advantageous as a surveillance strategy for tumor recurrence compared to imaging and CEA [43]. Further evidence for the need for greater sensitivity can be found in two studies on early stage lung cancer [9, 44] and a study on resectable colorectal cancer [45] in which patients found to be ctDNA negative using the currently available technologies had their disease relapse, suggesting that these technologies were not sufficiently sensitive to detect residual tumor in those patients.

**Figure 1:**
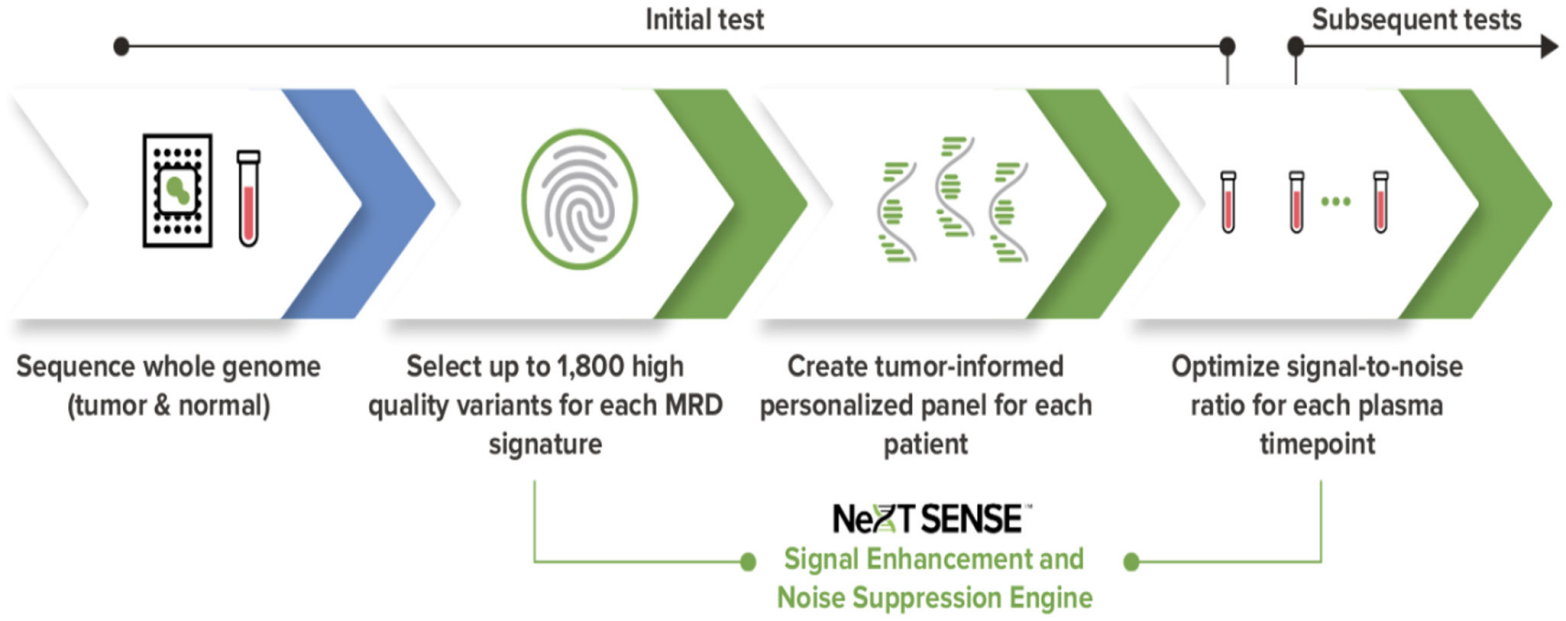
The NeXT Personal tumor-informed ctDNA detection process.

In this paper, we present the analytical validation of NeXT Personal, a tumor-informed, ctDNA assay (Figure 1) that utilizes whole genome sequencing (WGS) and NeXT SENSE™ (Signal Enhancement and Noise Suppression Engine) to significantly improve sensitivity to ctDNA, while preserving high specificity. Starting with whole genome sequencing of tumor and matched normal tissue samples from a patient, NeXT Personal designs a personalized panel of up to ~1,800 tumor variants, which enables enhanced sensitivity compared to other tumor-informed panel approaches that typically rely on up to ~50 whole exome sequencing (WES) based variants. This custom panel is then used to detect trace amounts of ctDNA from a patient’s blood sample for MRD or recurrence detection.

## RESULTS

### Overall performance

The performance of NeXT Personal was analytically validated by testing 123 tumor/normal/plasma sets from cancer patients, 131 donor normal plasma samples, and 2 tumor/normal matched cell line pairs, including a commercially available reference sample from SeraCare. For each sample set, a panel of up to ~1,800 tumor-specific variants was created from WGS data from the tumor tissue and the associated normal specimen. The analytical validation consisted of the following studies: accuracy, analytical range measurements (detection threshold, limit of blank, limit of detection, precision, linearity, limit of quantification, contrived sample functional characterization), clinical sample processing, specificity, effect of interfering substances, and effect of cfDNA input amount. The overall performance characteristics of the assay are summarized in Table 1.

**Table 1:** Performance specifications and analytical validation performance of NeXT Personal

Metric	Description	Measured performance
**Assay parameters**		
Panel size	Number of tumor-specific targets	Up to ~1,800 somatic variants
**Accuracy**		
Quantitative accuracy	Agreement between measured and target value	1.15 to 1,617 PPM (r^2^ = 0.9987)
Sensitivity	Rate of true positive detections of known positive samples	100% (98.5%–100%)
Positive predictive value (PPV)	Rate of true positive detections of total positive detections	100% (CI 96.0%–100%)
Negative predictive value (NPV)	Rate of true negative detections of total negative detections	100% (CI 98.5%–100%)
**Analytical range measurements**		
Detection Threshold^*^	Signal threshold for making a positive call	1.67 PPM
Limit of Blank^*^	Upper 95th percentile of signal on blank specimens	0.719 PPM
Limit of Detection^*^	Lowest concentration detectable in 95% of replicates	3.45 PPM
Precision	Coefficient of variation	4.15% (25,000 PPM) 3.55% (10,000 PPM) 12.8% (25 PPM)
Linearity	Range 0.8 to 300,000 PPM	Pearson correlation coefficient = 0.9998
Limit of Quantification	Lowest concentration measured with total error <25%	10 PPM
**Clinical sample processing**		
Sample processing success rate	Rate of success across 9 tumor types, stages I–IV	99.1%
**Specificity**		
Specificity	Rate of negative calls on normal samples	100% (CI 99.92%–100%)
**Effect of interfering substances**		
Genomic DNA (≥1.5 kb)	Consequence of leukocyte lysis in collected blood sample	Robust up to 25%
**Cell-free DNA input amount**		
Sample input quantity	Input range of clinical samples returning results within the defined total allowable error	2 to 30 ng

^*^Representative panel, performance of individual panels may vary. Abbreviation: CI: confidence interval.

### Accuracy

The accuracy study used an orthogonally confirmed, commercially available reference (Seraseq^®^ ctDNA MRD Panel Mix, SeraCare Life Sciences, Gaithersburg, MD) to assess the quantitative and qualitative accuracy of NeXT Personal. A personalized panel was created based on somatic variants detected in the WGS sequencing of the genomic DNA (gDNA) from the tumor and matched normal cell lines that were used to create the Seraseq control. A total of 137,532 somatic variants with allele frequency >10% were detected, of which approximately 1800 were selected to create the personalized panel. Target variants were chosen such that the distribution of single nucleotide variant (SNV) substitutions (e.g., C to T) as well as the allele frequencies mimicked those seen in typical clinical samples, and was necessary due to the atypical distribution of these features in the reference sample.

**Figure 2:**
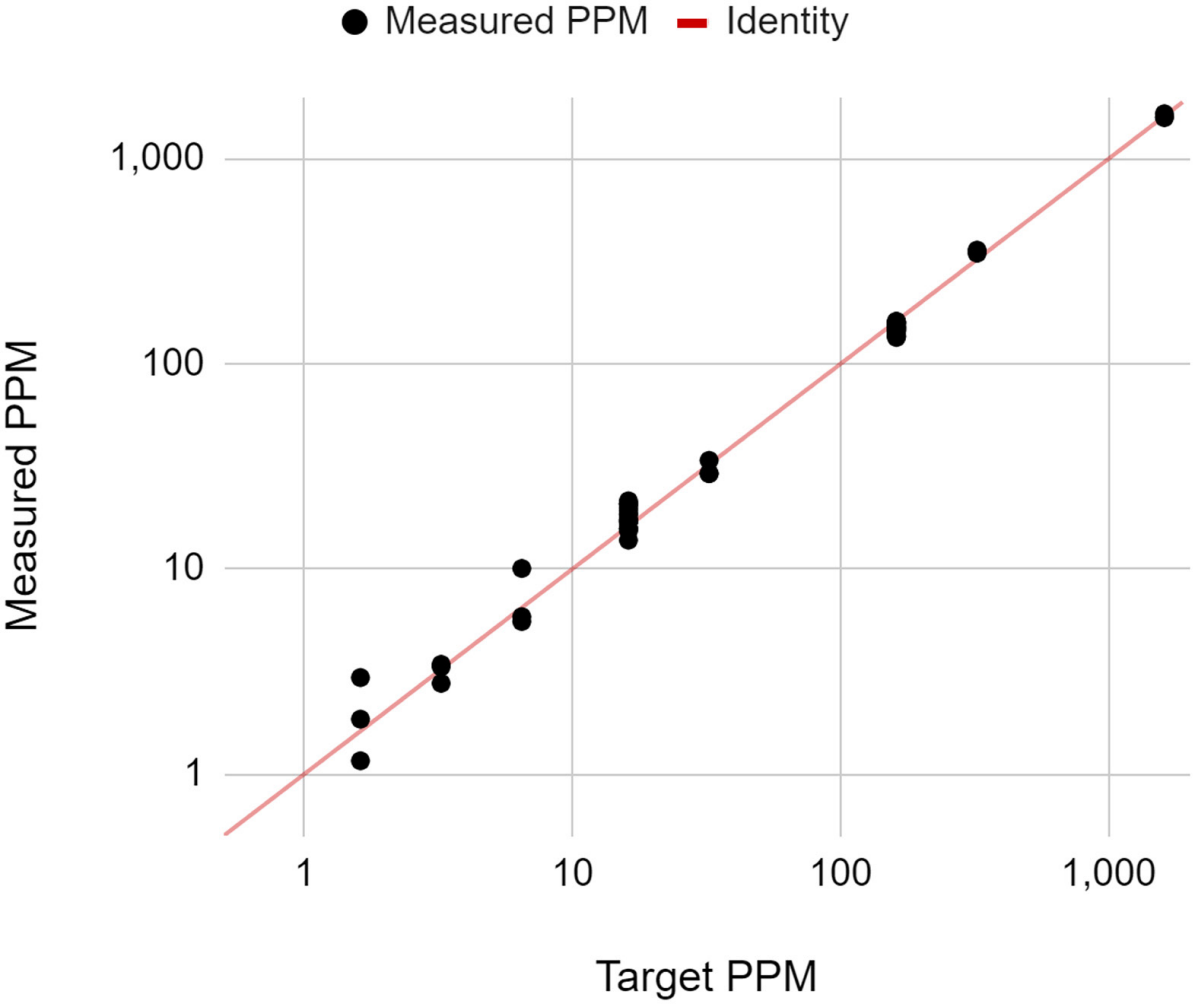
Measured ctDNA signal reported on NeXT Personal as a function of known ctDNA concentration. The solid line is the identity line (*y* = *x*).

To assess the quantitative accuracy of NeXT Personal over a range of ctDNA concentrations, samples of known concentration were prepared through a serial dilution of the Seraseq reference into its matched normal control. The dilution series consisted of 9 levels with ctDNA concentrations ranging from 1.15 to 1,617 PPM. At least 3 replicate samples per level were analyzed, with samples being processed by 2 operators. The ctDNA signal in each sample, as measured by NeXT Personal, is shown in Figure 2, as a function of known sample ctDNA concentration, with the identity relation shown by the solid line (*y* = *x*). Regression fit of the data yielded a correlation coefficient of 0.9987. Ordinary least squares regression was applied to the data. The 95% confidence interval (CI) for the intercept includes zero (−2.47, 3.25), and the CI for the slope includes unity (−0.99,1.01), indicating that the NeXT Personal measured results accurately reflect the known sample ctDNA concentration over the entire range of dilutions. Thus, NeXT Personal shows high analytical accuracy in measuring ctDNA concentration over that range.

To assess the qualitative accuracy of the assay, positive Seraseq reference samples with the lowest concentrations available commercially, along with negative samples, were tested with NeXT Personal. The known positive samples consisted of 20 replicates of the Seraseq 0.05% ctDNA control and 20 replicates of the Seraseq 0.005% ctDNA control, assayed with the personalized panel designed from WGS of the Seraseq tumor and matched normal cell lines. Negative samples included three replicates of the Seraseq 0% ctDNA control assayed with the personalized panel designed from WGS of the Seraseq tumor and matched normal cell lines, 46 normal cell line cell-free DNA (cfDNA) samples assayed with a personalized panel for the matched tumor cell line (see Materials and Methods), as well as 239 healthy donor normal clinical specimens assayed with a NeXT Personal panel designed for an unrelated cancer patient, for a total of 288 negative-control samples and 120 different panels. All samples were sequenced and analyzed with the NeXT Personal assay. The analysis assigns a *P*-value to describe the significance of the ctDNA measurement, and if the *P*-value is less than 0.001, then the sample is labeled as “ctDNA Positive”; otherwise it is labeled as “ctDNA Negative”. The results are summarized in Table 2. All positive and negative control samples were correctly classified by NeXT Personal.

**Table 2:** NeXT Personal accuracy

		Sample values	
		Positive	Negative
NeXT Personal	Positive	40	0
	Negative	0	288

Sensitivity, specificity, positive predictive value (PPV), and negative predictive value (NPV), were calculated as described in the Supplementary Materials, and the results are summarized in Table 3.

**Table 3:** NeXT Personal performance characteristics

Analytical sensitivity (95% CI)	100% (98.5%–100%)
Analytical specificity (95% CI)	100% (96.0%–100%)
Positive predictive value (95% CI)	100% (96.0%–100%)
Negative predictive value (95% CI)	100% (98.5%–100%)

### Analytical range measurements

NeXT Personal is a quantitative assay, providing a readout of the ctDNA signal of a plasma sample, in PPM. The analytical range of the assay helps users understand when multiple ctDNA measurements can be considered to be quantitatively different from each other, enabling quantitative monitoring of treatment response or disease burden.

To demonstrate the analytical range of NeXT Personal we used a contrived sample set created from cfDNA derived from a breast tumor cell line and a patient-matched normal cell line (see Materials and Methods). We created a NeXT Personal panel for these studies from WGS of the same tumor and normal cell line gDNA. The panel variants were verified to ensure that the SNV substitutions and allele frequencies closely mimicked a typical clinical sample’s panel. This NeXT Personal panel was used on the contrived sample set, as well as on a set of healthy donor normal samples, to measure the detection threshold, limit of blank (LOB), 95th percentile limit of detection (LOD_95_), limit of quantification (LOQ), precision, and linearity of the assay (see Discussion). While the detection threshold, LOB and LOD_95_ values are those of a representative panel and dependent on the panel used, the precision, linearity and LOQ can be considered as defining the performance of the assay itself, and are not dependent on individual panel designs.

### Detection threshold

For any given measurement, the detection threshold of NeXT Personal varies according to the parameters of the panel as well as the amount of cfDNA input into the assay. As stated above, measurements are called positive for ctDNA detection when the probability of the call having resulted from noise is <0.001, thus meeting our specificity requirement of >99.9%. As part of the NeXT Personal readout, we compute the ctDNA signal at the detection threshold. For the 190 measurements performed in the analytical range measurement studies, the detection threshold ranged from 1.47 to 1.87 PPM (1.67 PPM mean).

### Limit of blank

The limit of blank (LOB) for a ctDNA assay is the ctDNA noise signal measured in donor normal samples. For a tumor-informed assay such as NeXT Personal, this is appropriately characterized both for an individual panel, as described in this study, or for a set of panels, such as was done for the Specificity study described below. Characterization of the LOB for the contrived sample panel allows us to use the LOB measurement to derive the LOD_95_ in the subsequent study.

The limit of blank was measured using a collection of cfDNA from 121 donor normal plasmas and 46 normal cell line cfDNA samples (see Materials and Methods). The samples in this study were processed on different days, by different operators and using two reagent lots of the hybridization-capture enrichment kit (Twist Bioscience, South San Francisco, CA, USA). A Shapiro-Wilk test demonstrated significant departure of the data from normality and hence a non-parametric approach was taken. The results for two hybridization-capture enrichment kit lots are shown (Table 4). The higher value of the two results, 0.719 PPM, is taken as the LOB [46].

**Table 4:** Limit of blank (LOB) on two reagent lots

	Enrichment reagents lot A	Enrichment reagents lot B
Number of samples	82	85
Upper 95th percentile ctDNA signal of blank samples	0.493 PPM	0.719 PPM^*^

^*^The higher value of the two reagent lot results is taken as the limit of blank.

### Limit of detection

The limit of detection (LOD_95_) is defined as the ctDNA concentration at which 95% of measurements will yield a positive result [46]. In the LOD_95_ study, the contrived sample system was used to create a series of 7 low positive cfDNA samples with known ctDNA concentrations ranging from 0.8 to 8.2 PPM. Each of the 7 cfDNA samples was analyzed with NeXT Personal 5 times by 2 operators, for a total of 70 runs. To capture assay variability, the 5 replicate cfDNA libraries for each operator were enriched by the operator over 2 days, using different reagent lots of the hybridization-capture kit (Twist Bioscience) on each of the days. Using the precision profile approach [46], the LOD_95_ was calculated for each reagent lot (Table 5 and Supplementary Table 1). The final LOD_95_ determination is 3.45 PPM and is based on selecting the highest of the reagent lot estimates. Note that while this represents the lowest concentration at which 95% of positive samples are detected, any positive call above the detection threshold (~1.67 PPM for this panel) meets our specificity requirement, having less than 1 in 1000 probability of being a false positive.

**Table 5:** Calculated LOD for each reagent lot

	Enrichment reagents lot A	Enrichment reagents lot B
LOD (PPM)	1.80	3.45

### Precision

The goal of this study was to characterize the variability of the NeXT Personal ctDNA measurements among replicate samples at a range of potential signal levels. The total variance observed provides an estimate of how different two signals from a set of patient samples need be before they can be considered analytically different. Levels of 25, 1,000, and 25,000 PPM were selected as representative of low, medium, and high ctDNA signal levels in clinical samples (Supplementary Figure 1). Each of the three contrived cfDNA samples containing ctDNA at the selected signal levels was analyzed with NeXT Personal 24 times. To capture the most potential assay variability, the cfDNA samples were quantified by 2 operators, each using two different Cell-free DNA ScreenTape Analysis (Agilent Technologies) reagent lots prior to library preparation, and the pre-enrichment cfDNA libraries were enriched by 2 operators over 3 days using two different reagent lots of the hybridization-capture enrichment kit (Twist Bioscience). The overall precision was calculated as the coefficient of variation (CV), or the standard deviation divided by the mean value, over all runs for a given ctDNA concentration level.

A multivariate analysis of variance applied to the results did not point to any single factor as an overriding contributor to variability. The highest CV was observed at the lowest signal level (Table 6). The 95% confidence interval is 1.956 standard deviations of the measurement value, which at 25 PPM is 24.98%. Thus, we are using 25% as the total allowable error (TAE) for measurements in this study.

**Table 6:** Precision summary

Sample	Total runs	Mean concentration, measured (PPM)	Standard deviation (PPM)	Coefficient of variation (%)
High concentration (~25,000 PPM)	24	25,017	1,038.2	4.15%
Medium concentration (~1,000 PPM)	24	991.0	35.18	3.55%
Low concentration (~25 PPM)	24	26.02	3.323	12.77%

### Linearity

**Figure 3:**
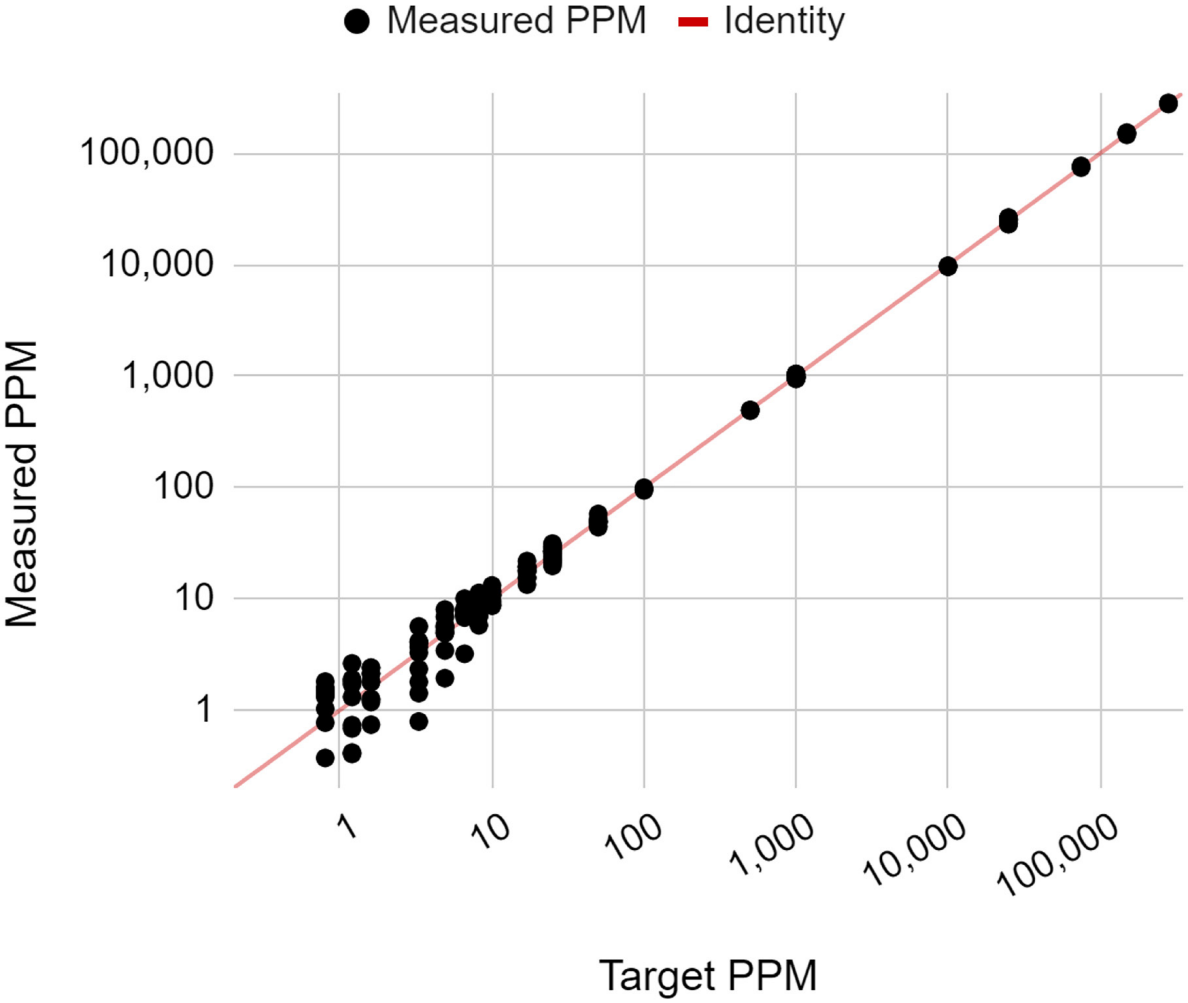
Measured ctDNA signal versus target ctDNA concentration (PPM), showing the linearity of the NeXT Personal assay.

The purpose of this study was to assess the linearity of the NeXT Personal assay. In order to determine how well the measured ctDNA signals correlate with the known ctDNA concentrations, a series of diluted samples were prepared at 19 different ctDNA concentrations ranging from 0.8 PPM to ~300,000 PPM. A minimum of 3 replicate samples at each ctDNA concentration level were tested with NeXT Personal, and the measured ctDNA signals are plotted against the known ctDNA concentration on a log-log scale, in Figure 3. The line shown is the identity line (*y* = *x*). A regression fit of the data yielded a Pearson correlation coefficient of 0.9998 (*p* < 0.001). This indicates that measured and input concentrations were linearly correlated over the range of 0.8 to 300,000 PPM.

### Limit of quantification

The limit of quantification (LOQ) is the lowest amount of a ctDNA signal that can be detected in a sample at a given level of accuracy. In this assay, we define the LOQ as the point at which a difference greater than 25% (the TAE) could be observed due to variability between two observations of the same ctDNA concentration. To determine the LOQ, 6 cfDNA samples were prepared using the contrived sample system to attain ctDNA concentrations of 6.6, 8.2, 10, 17, 25 and 50 PPM. Each of the 6 cfDNA samples was assayed 10 times with NeXT Personal. The 10 replicate cfDNA libraries at each ctDNA concentration were enriched by 2 operators over 2 days using different reagent lots of the hybridization-capture kit (Twist Bioscience) on each of the days. The mean of the measured ctDNA signal, in PPM, was compared to the target concentration, and the bias and standard deviations of the replicate measurements were also determined. The results are shown in Table 7. The lowest ctDNA concentration for which the total error is <25% for both reagent lots is chosen for the final LOQ, thus, the final LOQ was determined to be 10 PPM.

**Table 7:** Analysis of LOQ results by reagent lot (A, B)

Target PPM	Bias between measured and target PPM	Standard deviation of measured values (PPM)	Total Error (TE) in PPM (root mean square)	TE, %
50	0.87, −2	3.77, 3.78	3.86, 4.27	7.73%, 8.55%
25	0.64, 1.35	3.05, 2.83	3.11, 3.13	12.5%, 12.5%
17	1.78, −0.31	2.13, 2.35	2.78, 2.37	16.4%, 13.9%
**10**	0.74, 0.26	1.1, 1.73	1.33, 1.75	13.3%, 17.5%

### Contrived sample functional characterization

To demonstrate that the contrived cell line cfDNA samples used for the analytical range studies perform similarly to clinical samples, we conducted the contrived sample functional characterization study. In addition to the contrived samples described above, two sets of clinical cfDNA samples were prepared by serial dilution of ctDNA positive patient cfDNA with donor normal cfDNA to achieve samples with ctDNA concentrations ranging from 1.2 to 25 PPM. The clinical cfDNA samples originated from a breast cancer patient (Cureline, Brisbane, CA) and a non-small cell lung cancer patient (iProcess, Irving, TX, USA).

**Figure 4:**
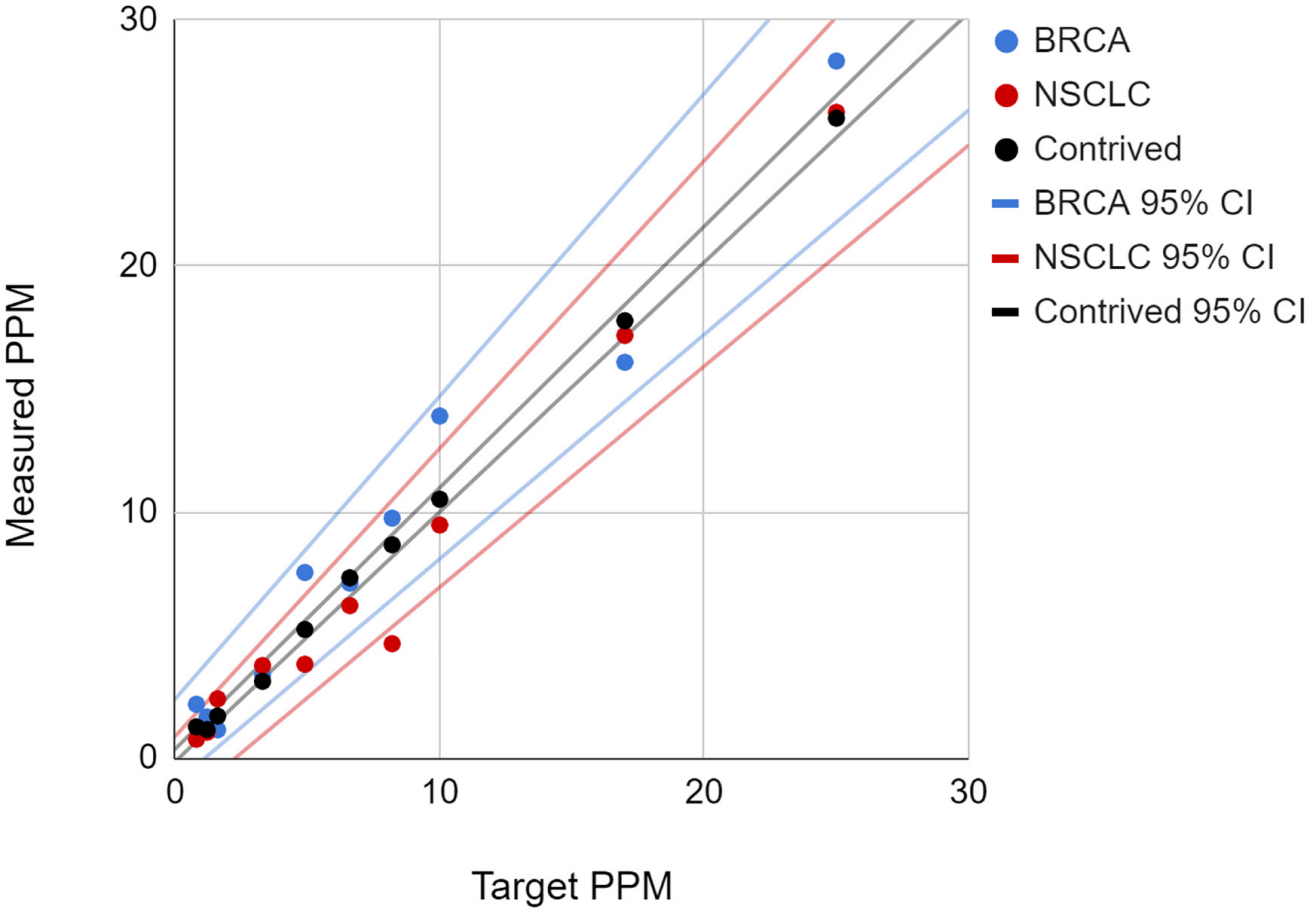
The plot of measured ctDNA concentration versus expected (target) concentration is shown for the contrived cell line and two clinical samples. The 95th percentile confidence intervals for the samples are shown as matching colored lines.

The contrived samples at each ctDNA concentration were assayed 10 times or more with NeXT Personal and the average ctDNA signal, in PPM, was plotted against the expected ctDNA concentration on the same x-y graph as the clinical samples, which were assayed a single time at each ctDNA concentration point (Figure 4).

To demonstrate equivalence, the slopes and intercepts of the regression fits were compared between the cell line samples and clinical samples. The results are summarized in Table 8. There is no significant difference between the contrived sample values and clinical sample values, with the 95% confidence intervals of the slopes and intercepts for both clinical samples fully encompassing the slope and intercept for the contrived sample. Based on the overlap in regression intercept and slope values of the contrived cell line and clinical samples, we conclude that the two types of samples are functionally comparable, and therefore the use of cell line samples in the validation study is appropriate.

**Table 8:** Linear regression slopes and intercepts from X-Y plots of measured concentration versus expected concentration

Sample	Linear regression intercept value (95% CI), PPM	Linear regression slope value (95% CI)
Contrived sample	0.120 (−0.14, 0.37)	1.04 (1.01, 1.06)
Breast Cancer (BC) Clinical sample	0.69 (−1.02, 2.41)	1.07 (0.91, 1.23)
Lung Cancer (LC) Clinical sample	−0.57 (−2.03, 0.89)	1.03 (0.90, 1.17)

### Clinical sample processing

The purpose of this study was to demonstrate the end-to-end processing success rate for NeXT Personal using clinical samples. This study was conducted using clinical specimen sets from various vendors (see Materials and Methods) for which tumor tissue, normal tissue, and a plasma sample were available. A total of 118 clinical sample sets were assayed, representing nine different cancer types (Table 9). Two specimen sets were found to be from the same patient, resulting in 117 unique patients in the dataset.

**Table 9:** Clinical samples used in the NeXT Personal clinical sample processing study

Type	Stage				Type total (%)
	I	II	III	IV	
Lung	4	10	6	1	28 (23.7%)
Colorectal	5^*^	7	12	0	24 (20.3%)
Bladder	3	7	8	4	22 (18.6%)
Breast	9	12	6	1	21 (17.8%)
Melanoma	1	3	5	0	9 (7.6%)
Prostate	0	3	1	2	6 (5.1%)
Ovarian	0	1	2	0	3 (2.5%)
Renal	0	0	2	1	3 (2.5%)
Stomach	0	0	2	0	2 (1.7%)
Stage Total (%)	22 (18.6%)	43 (36.4%)	44 (37.3%)	9 (7.6%)	118 (100%)

^*^Two specimen sets were found to be from the same patient.

Assay variability was captured by using two different reagent lots for the Cell-free DNA ScreenTape Analysis (Agilent Technologies) for cfDNA quantification prior to library preparation, and two different reagent lots of the hybridization-capture enrichment kit (Twist Bioscience) for cfDNA library enrichment with the tumor-informed panels. Furthermore, both of these sample processing steps were performed by 2 operators over multiple days. All 118 tumor samples had >20% tumor content, as determined by pathologists. Macrodissection of the FFPE sections was performed prior to genomic DNA extraction when it could increase the tumor content of the resulting sample. All tumor samples yielded sufficient DNA to move forward with WGS and NeXT Personal panel design.

**Figure 5:**
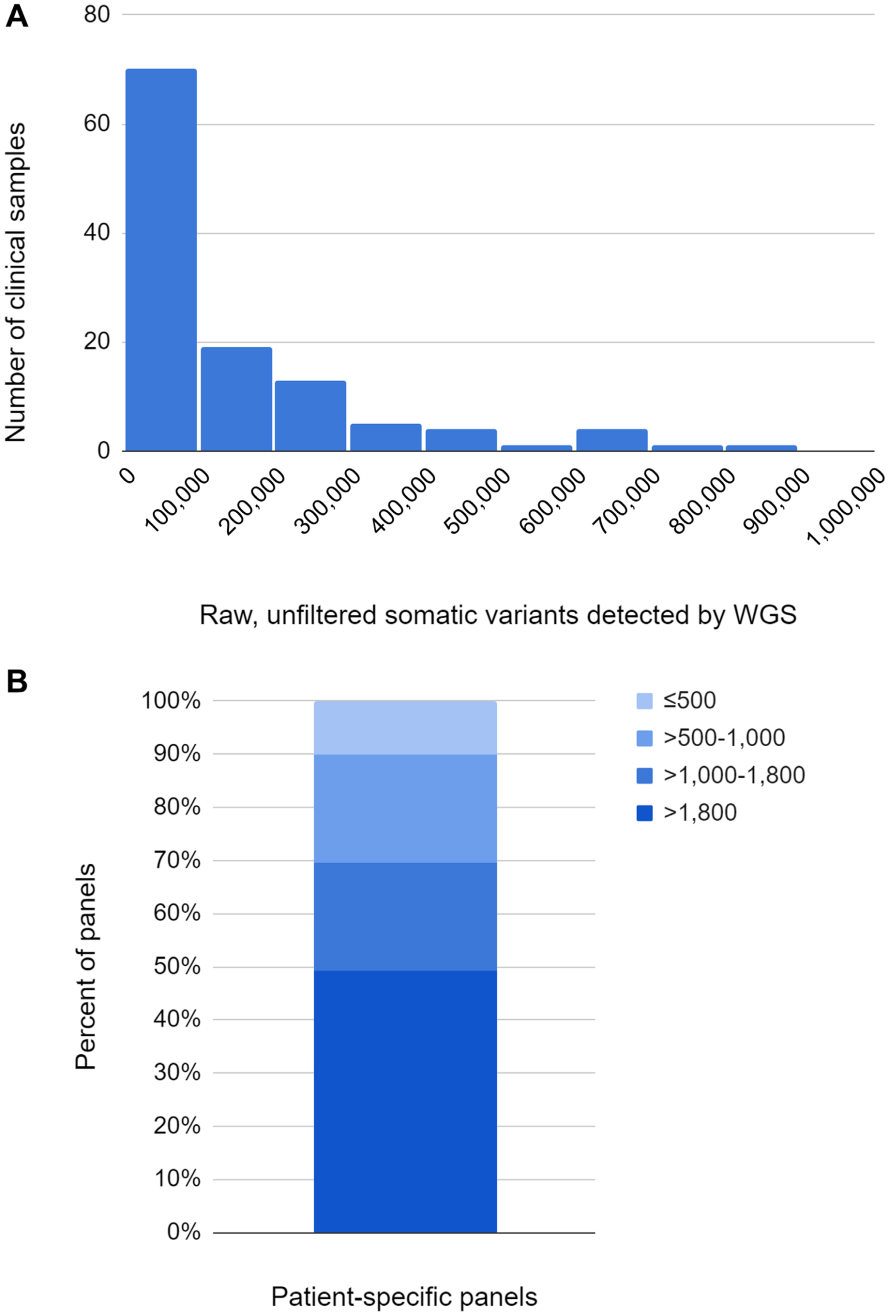
(**A**) Number of raw, unfiltered somatic variants detected by WGS. (**B**) Number of somatic variant targets per panel.

For over half of the 118 clinical sample sets processed, WGS of the tumor and associated normal sample resulted in 100,000 or fewer raw, unfiltered somatic variant calls (Figure 5A). Nearly half of the resulting 118 patient-specific panels had >1,800 somatic variant targets, with 70% of the panels having >1,000 somatic variant targets and over 85% having >500 somatic variant targets (Figure 5B). These panels had a median detection threshold of 2.0 PPM (range 1.0 - 12 PPM). Extraction of cfDNA from up to 4.1 mL (minimum 2.1, median 3.5 mL) of plasma produced sufficient cfDNA for analysis (range 3.4 to 420 ng). Of the 118 clinical specimen sets, 5 had plasma that did not match the provided tumor/normal tissue samples. As all evidence suggested a vendor mistake, these 5 cases were excluded from the study. One plasma sample was failed due to detection of a contaminant in the sample. Therefore, in this study, the processing success rate was 99.1% (112 samples out of 113 were successfully processed).

### Specificity

**Figure 6:**
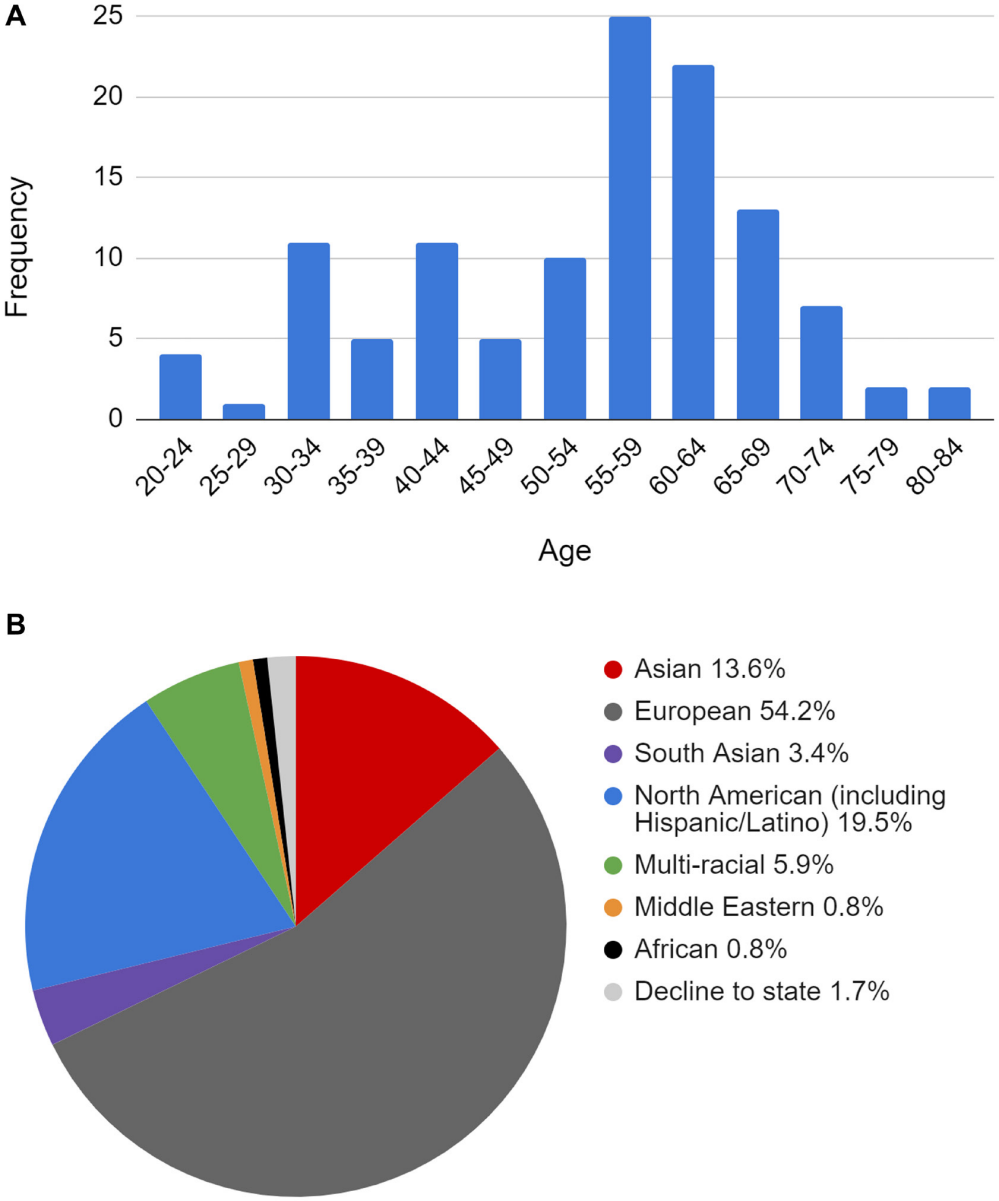
(**A**) Age distribution of donor normal samples used in specificity studies. (**B**) Self-reported demographics of donor normal samples used in specificity studies.

In the specificity study, normal plasma samples (“blank” samples) were assayed with the NeXT Personal panels described in the clinical sample processing study. Normal blood samples were collected in Streck cfDNA tubes from 118 donors at the Stanford Blood Center (Stanford, CA) and processed to plasma for use in these studies. Each of the 118 donor cfDNA samples was enriched with a different, unrelated patient panel to detect the presence of patient-specific tumor variants. Figure 6 describes the characteristics of the normal blood donors.

Per industry convention [46], sample processing for this study was performed by 2 operators over multiple days using two different reagent lots for cfDNA quantification and library enrichment. Specificity was 100%, as measured by this study (95% CI 96.8 to 100%) (Table 10).

**Table 10:** Specificity results

Approach	Panels tested	Specificity (95% CI)
Empirical measure with donor normals	118	100% (96.8%, 100%)
In silico flanking	23,600	99.98% (99.96%, 100%)
*In silico* reshuffling	23,600	99.95% (99.92%, 99.98%)

Two *in silico* approaches were used to more precisely describe the specificity of the assay, the method of Abbosh et al. (2023) [44] that draws on loci flanking each targeted site and an internally developed method relying on somatic variant target reshuffling (see Materials and Methods). These analyses each resulted in 23,600 *in silico* panels with the detection of 4 and 31 false positives, respectively, giving measured specificities of 99.98% (95% CI 99.96% to 100.00%) and 99.95% (95% CI 99.92% to 99.98%) (Table 10).

### Interfering substances

#### Genomic DNA

The most common contaminant that could interfere with the NeXT Personal assay is genomic DNA (gDNA) from leukocyte lysis, which can occur at any point between blood collection and cfDNA extraction. While gDNA is usually observed to be quite high in molecular weight (>10 kb), in this study we chose to look at a contaminating gDNA species around 1.5 kb, which represented the shortest gDNA species observed in over a hundred patient cfDNAs extracted from plasma following Personalis standard operating procedures. The contaminating gDNA in this study was produced through enzymatic digestion using the KAPA Frag kit (Roche Sequencing Solutions, Pleasanton, CA) from high molecular weight gDNA from NA12878 (Coreill, Camden, NJ, USA). Contrived cell line cfDNA samples containing ctDNA levels of 25, 1,000, and 25,000 PPM were spiked with 25% gDNA, or used without contaminating gDNA as control. Note that 25% represented the highest level of contamination observed in the aforementioned patient plasma samples. Samples were tested on NeXT Personal in replicates of 5. The mean concentration differences between spiked and unspiked samples are summarized in Table 11.

**Table 11:** Effect of gDNA spiking on ctDNA signal measurements

Concentration level	Mean difference between gDNA-spiked and unspiked groups (%)
High concentration (25,000 PPM)	−9.33%
Medium concentration (1,000 PPM)	−4.29%
Low concentration (25 PPM)	−8.47%

These mean differences fall below the variance of 12.77% observed in the precision study, suggesting a limited impact on determinations of ctDNA concentration due to the presence of gDNA in the sample.

In addition to gDNA contamination, we investigated the effects of hemoglobin contamination at 4x the allowable limit for laboratory processing [47] and the carryover of the final wash buffer used in cfDNA extraction at concentrations higher than expected to be present. Neither of these showed significant effects (Supplementary Figures 2 and 3).

### Effect of cfDNA input amount on ctDNA measurements

**Figure 7:**
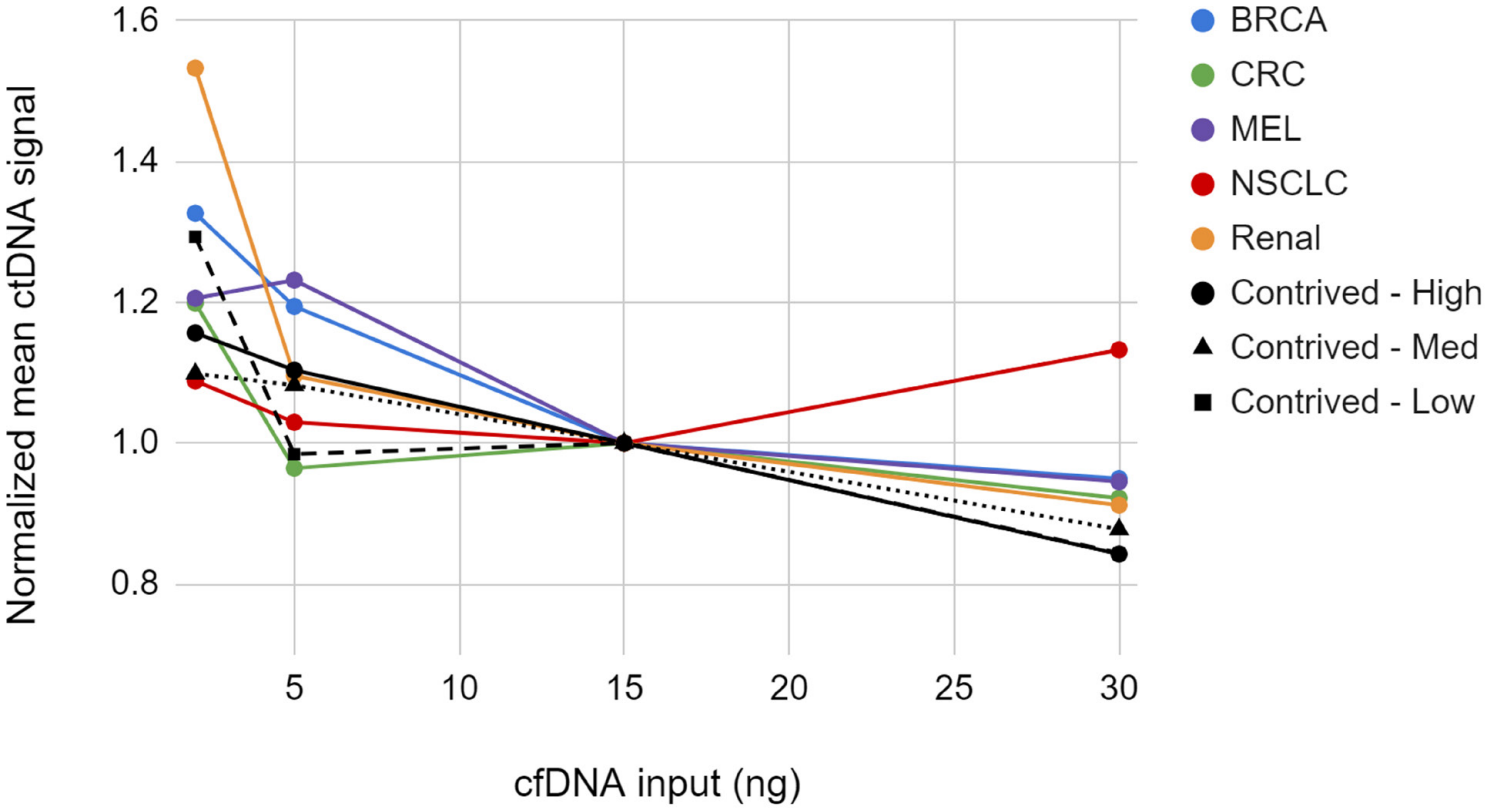
Normalized mean ctDNA signal for 5 cancer samples and 3 contrived samples at various cfDNA input quantities. Points represent the mean of replicates normalized to the 15 ng input level.

We examined the impact of cfDNA input amount on the measured ctDNA signal, in PPM. In these studies we titrated the cfDNA input from 2 to 30 ng. The modal cfDNA input for the NeXT Personal assay is approximately 15 ng cfDNA and therefore this amount was chosen as the input level for comparison. Clinical cfDNA samples from patients with breast cancer, colorectal cancer, non-small cell lung cancer (NSCLC), melanoma, and renal cancer were diluted to a ctDNA concentration of approximately 25–30 PPM and tested in triplicate with NeXT Personal. The contrived sample system was used to examine cfDNA input across the same range at 25, 1000, and 25,000 PPM measured 5 times at each point. Figure 7 and Supplementary Table 2 show the NeXT Personal results from this study.

We observed that the measured ctDNA signal trends upward as the cfDNA input amount decreases. For the contrived samples, only the 2 ng cfDNA input samples showed significant deviation from the standard 15 ng input samples. Similarly, for the clinical samples, the 2 ng input differed significantly from the 15 ng input for the breast cancer, melanoma, and renal cancer samples. The deviation of all clinical sample points in the 2 to 30 ng range fell within the TAE (see Supplementary Materials). From this study we conclude that the measured ctDNA signal is not significantly affected over an input range of 5 to 30 ng.

## DISCUSSION

Effective clinical detection of ctDNA is dependent upon a sensitive and highly specific quantitative diagnostic assay with robust analytical performance. We demonstrate the analytical performance of NeXT Personal in a comprehensive manner, characterizing the limit of blank (LOB), limit of detection (LOD_95_), limit of quantification (LOQ), precision, and linearity, as well as demonstrating the specificity of the assay on a large set of clinical samples.

A commercially available control along with a large set of clinical normal samples were used to establish the overall quantitative and qualitative accuracy of the assay. The ctDNA measurements showed excellent agreement to known ctDNA concentrations over a range of 1.15 to 1,617 PPM (regression correlation coefficient of 0.9987) and the sensitivity, specificity, PPV, and NPV were measured to be 100%. Two *in silico* approaches to precisely define specificity agreed well with our targeted specificity of 99.9%, which is enabled by our proprietary noise reduction algorithms.

**Figure 8:**
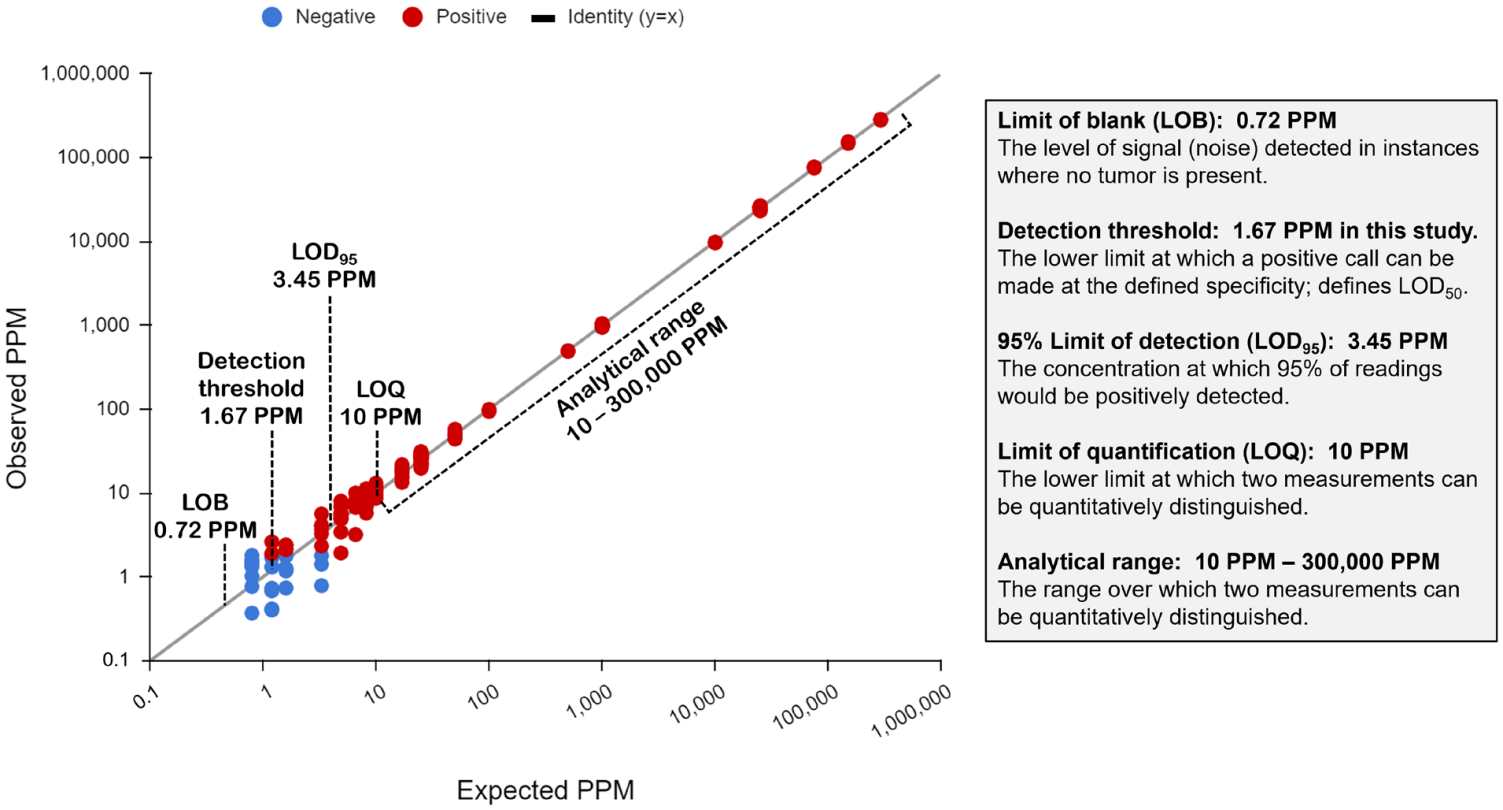
Major analytical performance measurements of the NeXT Personal assay.

A contrived sample system was used to characterize the analytical range of the assay (Figure 8), demonstrating the capability of the assay to make quantitative determinations of differences in the concentrations of ctDNA present. An overview of the analytical range results are shown in Figure 8. The LOB was determined to be 0.719 PPM, established by processing 167 blank samples. The detection threshold at 99.9% specificity for these studies (1.67 PPM) was similar to that observed in the clinical sample set (median 2.0 PPM). The LOD_95_ is 3.45 PPM. This high level of sensitivity holds the promise of earlier detection of residual or recurrent disease and potentially better outcomes for the patient.

Importantly, the detection of ctDNA by NeXT Personal has been shown to be linear over a wide range, from 0.8 to 300,000 PPM. Assay precision ranged from a coefficient of variation (CV) of 3.6% to 12.8%, with the highest CV at the lowest ctDNA concentration. We established a total allowable error (TAE) of 25% based on our precision measurements. We then determined the LOQ, the “lowest amount of a measurand in a material that can be quantitatively determined with stated accuracy (i.e., the total allowable error (TAE))” [46]. This resulted in an LOQ of 10 PPM. This robust determination of LOQ allows calls above 10 PPM, differing by more than 25%, to be considered analytically different.

The assay was shown to perform well with clinical samples, across a range of tumor types and stages, as well as cfDNA input amounts, with a sample processing success rate of 99.1%. Further, the assay retains its quantitative capabilities across the anticipated range of cfDNA inputs.

NeXT Personal’s tumor-informed ctDNA detection approach, combining whole genome sequencing with NeXT SENSE, results in an ultra-sensitive and highly specific quantitative diagnostic assay. We have demonstrated the analytical performance of NeXT Personal across all critical aspects of a ctDNA assay.

The high level of sensitivity and specificity of NeXT Personal has the potential to improve both lead times to recurrence detection and detection of molecular residual disease. The quantitative aspect of the assay is a differentiating feature which can enable more robust disease and treatment monitoring. These results suggest strong potential for clinical use of the assay in ctDNA monitoring of solid tumor cancers.

## MATERIALS AND METHODS

The accuracy, specificity, analytical range measurements and clinical sample processing studies were conducted in the Clinical Laboratory Improvement Amendments (CLIA)-certified and College of American Pathologists (CAP)-accredited laboratories at Personalis, Inc., as guided by the Association for Molecular Pathology (AMP) and CAP’s joint recommendations and following the standard operating procedures at Personalis. Interfering substances and cfDNA input amount experiments were conducted in the Personalis R&D laboratories and followed similar procedures and practices.

### Sample acquisition

Healthy donor plasma was obtained from the Stanford Blood Center (Stanford, CA, USA). Matched sets of patient FFPE tumor tissue, buffy coat or adjacent normal FFPE tissue, and plasma were sourced from BOCA Biolistics (Pompano Beach, FL, USA), BioOptions (Brea, CA, USA), Cureline (Brisbane, CA, USA), Discovery Life Sciences (Huntsville, AL, USA), DxBioSamples (San Diego, CA, USA), and Us4Cure (Agoura Hills, CA, USA). Cell lines were purchased from the American Type Culture Collection and maintained under the recommended conditions. The commercially available MRD control (Seraseq ctDNA MRD Panel Mix, LGC SeraCare) was sourced directly from LGC SeraCare (Milford, MA, USA).

### NeXT Personal probe panel design

NeXT Personal custom probe panels were designed from WGS of matched tumor and normal samples using the NeXT Personal platform. Purchased FFPE tissues and buffy coat samples were processed in the Personalis CLIA/CAP laboratories following the standard operating procedures at Personalis. Briefly, one hematoxylin and eosin (H&E) stained slide was prepared from each FFPE block and used to determine the tumor content of the tissue sample. For tumor samples, macrodissection was performed when it could increase the tumor content of the resulting sample. Genomic DNA was extracted from the tissue sections and the buffy coat samples using commercially available kits (QIAGEN, Germantown, MD, USA). DNA libraries were prepared with 50 ng to 500 ng of acoustically sheared genomic DNA (Covaris LLC, Woburn, MA, USA) using a commercially available KAPA Kit (Roche Sequencing Solutions, Pleasanton, CA, USA) and Personalis-optimized workflows. Whole genome sequencing was performed using NovaSeq instruments (Illumina, San Diego, California) to a depth of at least 30x. Somatic variant calling, ctDNA target selection, and probe design were completed using the proprietary algorithms of the NeXT Personal platform. Briefly, following somatic variant calling, the algorithms exclude variants with an allele frequency less than 10% and/or located in problematic genomic regions, such as regions with known germline and clonal hematopoiesis of indeterminate potential (CHIP) variants, high GC content (80% and above) and low sequence complexity. Next, the remaining somatic variants are ranked by the tumor allele frequency and the error-rate of the substitution. Up to ~1,800 personalized panel targets are selected genome-wide from the high-quality and low-noise somatic variants for inclusion in the NeXT Personal panel.

### Preparation of contrived cell line cfDNA samples

The HCC1954 (CRL-2338, stage IIA, invasive breast ductal carcinoma) and HCC1954BL (CRL-2339, B lymphoblast) patient-matched cell lines were maintained in RPMI 1640 media with 10% Fetal Bovine Serum. These cells were incubated in a proprietary media system prior to the collection of cfDNA from the media. Extraction of cfDNA from the cell culture media was performed using a commercially available kit (QIAGEN, Germantown, MD, USA) and size selected with AMPure XP beads (Beckman Coulter, Indianapolis, IN) to recapitulate the cfDNA fragment sizes observed in patient plasma samples. Quantification of the cfDNA was performed with the Qubit dsDNA BR assay (Thermo Fisher Scientific, Fremont, CA, USA) and Cell-free DNA ScreenTape Analysis (Agilent Technologies, Santa Clara, CA, USA). Subsequently, cfDNA derived from HCC1954 was serially diluted with cfDNA derived from HCC1954-BL. Except as noted, all pre-enrichment libraries were created with 15 ng of cfDNA.

### Preparation of patient and healthy honor cfDNA samples

Plasma processing and cfDNA extraction was performed according to the standard operating procedures at Personalis. Healthy donor plasma was isolated from whole blood collected in Streck Cell-Free DNA BCTs (Streck, La Vista, NE, USA) and clarified using sequential centrifugation at 1,600 RCF and 16,000 RCF, respectively. Patient plasma from vendors was thawed and then clarified at 15,000 RCF prior to cfDNA extraction. For all plasma samples, cfDNA was extracted from up to 4.1 mL clarified plasma using a commercially available kit (QIAGEN, Germantown, MD, USA) and quantified using Cell-free DNA ScreenTape Analysis (Agilent Technologies, Santa Clara, CA, USA). For the clinical sample processing and specificity studies, up to 30 ng cfDNA was included in the preparation of pre-enrichment libraries. For the contrived sample functional characterization study, patient cfDNA was serially diluted with a healthy donor cfDNA sample, and then pre-enrichment libraries were created using 15 ng of cfDNA.

### NeXT Personal cfDNA library preparation, target enrichment and sequencing

Library preparation, target enrichment and sequencing of the cfDNA samples was performed according to the standard operating procedures at Personalis. Briefly, pre-enrichment libraries were prepared from 2 ng to 30 ng cfDNA input using a commercially available KAPA Kit (Roche Sequencing Solutions, Pleasanton, CA, USA) and Personalis-optimized workflows. A Lunatic spectrophotometer (Unchained Labs, Pleasanton, CA, USA) was used to quantify the pre-enrichment libraries. Up to 1500 ng of library DNA was enriched with NeXT Personal custom probe panels using proprietary modifications to a commercially available Twist kit and hybridization-capture workflow (Twist Bioscience, South San Francisco, CA, USA). The post-enrichment libraries were significantly over-sequenced on NovaSeq instruments (Illumina, San Diego, CA, USA) in order to optimize the number of unique molecules observed.

### NeXT Personal MRD analysis

The results and data presented in this study utilize the production version of the NeXT Personal analysis pipelines, in use for the LDT version of the assay at the time of publication. Briefly, sequencing data from cfDNA libraries enriched with patient specific panels is used to form consensus reads of the captured molecules across the targeted regions. Proprietary grouping and filtering approaches ensure the formation of accurate read groupings prior to molecular consensus formation. Following noise suppression, the individual ctDNA signals were aggregated across the targets in each panel to obtain the total ctDNA signal. The ctDNA signal is reported in PPM, calculated as the number of consensus molecules containing a tumor derived variant divided by the total number of consensus molecules covering MRD targets.

Statistical analysis is undertaken to vet and aggregate the ctDNA signal across all MRD targets. To determine ctDNA detection status, a one-tailed Poisson test was performed based on the hypothesis that the observed signal comes from noise. The observed aggregate ctDNA signal for each panel served as the tested value, and the expected noise arising from the accumulated background error serves as the mean of the Poisson distribution. A ctDNA positive call is made only when the *p*-value assigned by that statistical analysis is less than 0.001 (indicating a false positive rate of less than 1 in 1,000).

### Specificity evaluation using simulated panels

The specificity of the NeXT Personal platform was empirically examined using the 118 patient-specific panels described in the Clinical Sample Processing study (see Results). Two complementary analyses were performed *in silico* with 23,600 simulated panels each. First, the simulated panels were generated according to the approach of Abbosh et al. 2023 [44]. In brief, *in silico* panels were created via replacement of the original MRD variants in the 118 patient-specific panels, maintaining the size and trinucleotide error context of each panel. MRD targets were replaced by random sampling with bases within 50 bp of the actual target with similar coverage, passing NeXT Personal target selection requirements, and not indicated as known germline variants in dbSNP (build 146, https://www.ncbi.nlm.nih.gov/pmc/articles/PMC102496/). Sequencing data from the Specificity study was then processed through the standard NeXT Personal pipeline to detect any false positive calls. Second, the simulated panels were generated by re-shuffling the targets used across all panels in the Specificity study into new combinations that maintain the size and trinucleotide error context of each panel. Sequencing data for the new combinations of targets from the Specificity study was then re-processed through the standard NeXT Personal pipeline to detect any false positive calls.

### Statistical analysis

Analysis was performed in R Statistical Software. All statistical tests were 2-sided unless otherwise stated. Detailed descriptions of the statistical analyses can be found in the Supplementary Materials.

## SUPPLEMENTARY MATERIALS


