# Correction: Analytical validation of NeXT Personal^®^, an ultra-sensitive personalized circulating tumor DNA assay

**DOI:** 10.18632/oncotarget.28581

**Published:** 2024-06-20

**Authors:** Josette Northcott, Gabor Bartha, Jason Harris, Conan Li, Fabio C.P. Navarro, Rachel Marty Pyke, Manqing Hong, Qi Zhang, Shuyuan Ma, Tina X. Chen, Janet Lai, Nitin Udar, Juan-Sebastian Saldivar, Erin Ayash, Joshua Anderson, Jiang Li, Tiange Cui, Tu Le, Ruthie Chow, Randy Jerel Velasco, Chris Mallo, Rose Santiago, Robert C. Bruce, Laurie J. Goodman, Yi Chen, Dan Norton, Richard O. Chen, John M. Lyle

**Affiliations:** ^1^Personalis, Inc., Fremont, CA 94555, USA; ^*^Co-last authors

**This article has been corrected:** The following author has been added to the author list:

Massimo Morra

^1^Personalis, Inc., Fremont, CA 94555, USA

Original article: Oncotarget. 2024; 15:200–218. 200-218. https://doi.org/10.18632/oncotarget.28565

