# Supplementary material for: Analytical validation of NeXT Personal®, an ultra-sensitive personalized circulating tumor DNA assay
Source: Oncotarget. 2024 Mar 14;15:200–18. doi: 10.18632/oncotarget.28565 (PMC10939476; doi:10.18632/oncotarget.28565)
Supplement: Supplementary file 1 [file oncotarget-15-28565-s001.pdf]

Analytical validation of NeXT Personal®, an ultra-sensitive personalized circulating tumor DNA assay

SUPPLEMENTARY MATERIALS

Supplementary Results

Interfering substances: hemoglobin

Another common contaminant in the plasma fraction is hemoglobin, released by the hemolysis of red blood cells. To study the effect of hemoglobin on the NeXT Personal assay, hemoglobin was added into normal blood from three donors at 0, 0.5, 1, 2, and 4 mg/mL. The highest amount (4 mg/mL) corresponds to nearly 3% hemolysis, and is 4 times as high as the allowable limit for laboratory processing [47]. After hemoglobin addition, the blood samples were processed according to the standard operating procedures for the NeXT Personal assay.

Measurements of hemoglobin before and after cfDNA extraction showed that hemoglobin was largely removed during extraction, resulting in no effect on cfDNA extraction or library yields, even at the highest amount of hemoglobin addition (Supplementary Figure 2). We conclude that even with the presence of substantial free hemoglobin present in the patient blood donor sample, the NeXT Personal assay is not materially affected.

Interfering substances: nucleic acid extraction wash buffer

A risk-based assessment of the overall NeXT Personal lab process identified contamination of extracted cfDNA by the wash buffer from the step prior to elution as the highest potential risk for interfering substances resulting from the protocol. To demonstrate the robustness of the assay to this potential interferent, we added this wash buffer into purified cfDNA at a range of concentrations, spanning and exceeding those considered likely to occur. As shown in Supplementary Figure 3, there was no impact on cfDNA library yield due to the presence of this potential interferent. Sequencing of the libraries showed no significant changes in error rates in the presence of the interferent (data not shown).

For the melanoma sample, the mean ctDNA signal at 5 ng input was significantly higher than at 15 ng input, but this difference was within the 25% TAE of our analytical range. In the NSCLC sample, the mean ctDNA signal at 30 ng input was also significantly higher than at 15 ng input, but we attribute this to the atypically small standard deviation (CV = 2.6%) associated with those replicates. The actual deviation, 13.0%, is well within the 25% allowable error.

Supplementary Methods

Accuracy calculation

For the accuracy study, the reported confidence interval (CI) is the 95% CI, which is equivalent to 1.956 standard deviations above and below the mean. The assay sensitivity was calculated as the number of true-positive results divided by the sum of true-positive and false-negative results; assay specificity was calculated as the number of true-negative results divided by the sum of true-negatives and false-positive results; the positive predictive value was calculated as the number of true-positive results divided by the sum of true-positive and false-positive results; and the negative predictive value was calculated as the number of true-negative results divided by the sum of false-negative plus true-negative results.

$$Sensitivity = \frac{True\ Positives\ (TP)}{True\ Positives\ (TP) + False\ Negatives (FN)} = \frac{40}{40+0} = 100\%$$

$$Specificity = \frac{True\ Negatives\ (TN)}{True\ Negatives\ (TN) + False\ Positives\ (FP)} = \frac{288}{288+0} = 100\%$$

$$Positive\ Predictive\ Value\ (PPV) = \frac{TP}{TP+FP} = \frac{40}{40+0} = 100\%$$

$$Negative\ Predictive\ Value\ (NPV) = \frac{TN}{TN+FN} = \frac{288}{288+0} = 100\%$$

Limit of detection calculation

The limit of detection (LOD_{95}) is defined by convention as the ctDNA concentration at which 95% of measurement results give a positive outcome [46]. The LOD_{95} is reported in units of parts per million (PPM).

In this LOD study, the contrived sample system was used to create a series of 7 low positive samples with known tumor mutation concentrations ranging from 0.8 PPM to 8.2 PPM. Each sample was run 5 times by 2 operators, for a total of 70 runs. The 5 replicates were enriched by the operator over 2 days, using different lots of the hybridization-capture enrichment kit.

The results for each reagent lot were plotted as the standard deviation (SD) of ctDNA signal levels versus the measured ctDNA signal. Using the precision profile approach [46], a third order polynomial equation was fit to each plot, and the best fit coefficients B_1, B_2, B_3, and B_4 were obtained (Eq. 1).

$$SD = B_1 + B_2X + B_3X^2 + B_4X^3 \quad \text{(Eq. 1)}$$

where the polynomial coefficients were obtained as shown in Supplementary Table 2.

Using Eq. 2, a trial LOD was calculated for mean detection threshold and then numerically iterated to the new value until the result converged.

$$LOD = LOB + 1.645 \cdot (B_1 + B_2 \cdot LOD + B_3 \cdot LOD^2 + B_4 \cdot LOD^3), \quad \text{(Eq. 2)}$$

In Eq. 2, 1.645 represents the shift that would put 95% of the normal distribution of signals above the LOB (0.72 PPM per above) when the SD = 1. The actual SD can be found from Eq. 1 using the LOD for X. Since the highest LOD for the reagent lots is chosen for the final LOD, the final LOD is 3.45 PPM.

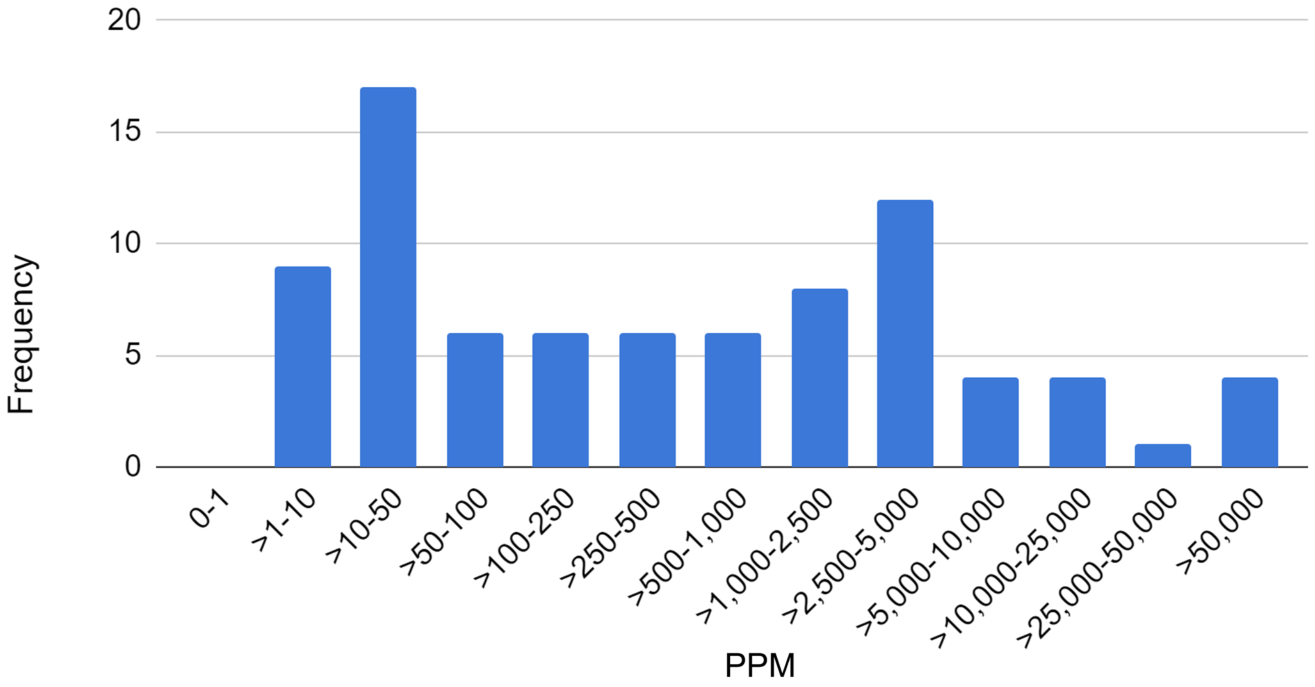

Supplementary Figure 1: Histogram of the ctDNA signals (PPM) for clinical samples with NeXT Personal positive ctDNA calls. Levels of 25, 1,000, and 25,000 PPM were selected for the precision study as they are representative of low, medium, and high positive signals observed in clinical samples.

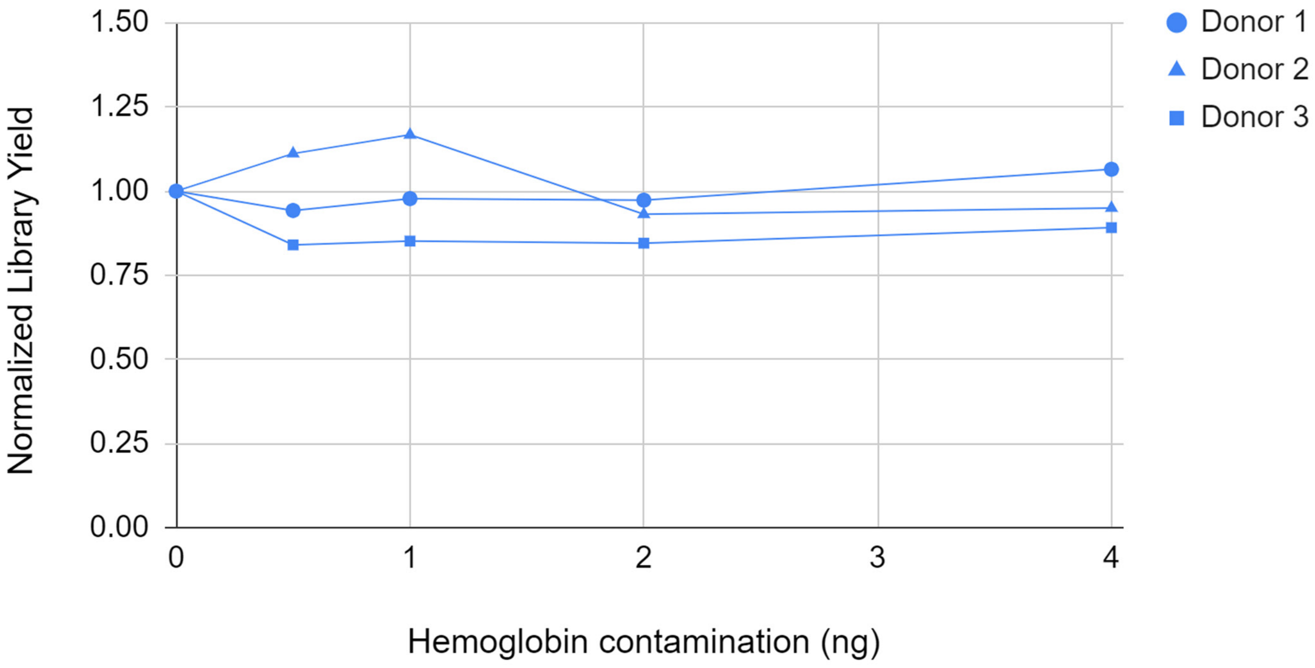

Supplementary Figure 2: Impact of hemoglobin contamination on cfDNA library yield.

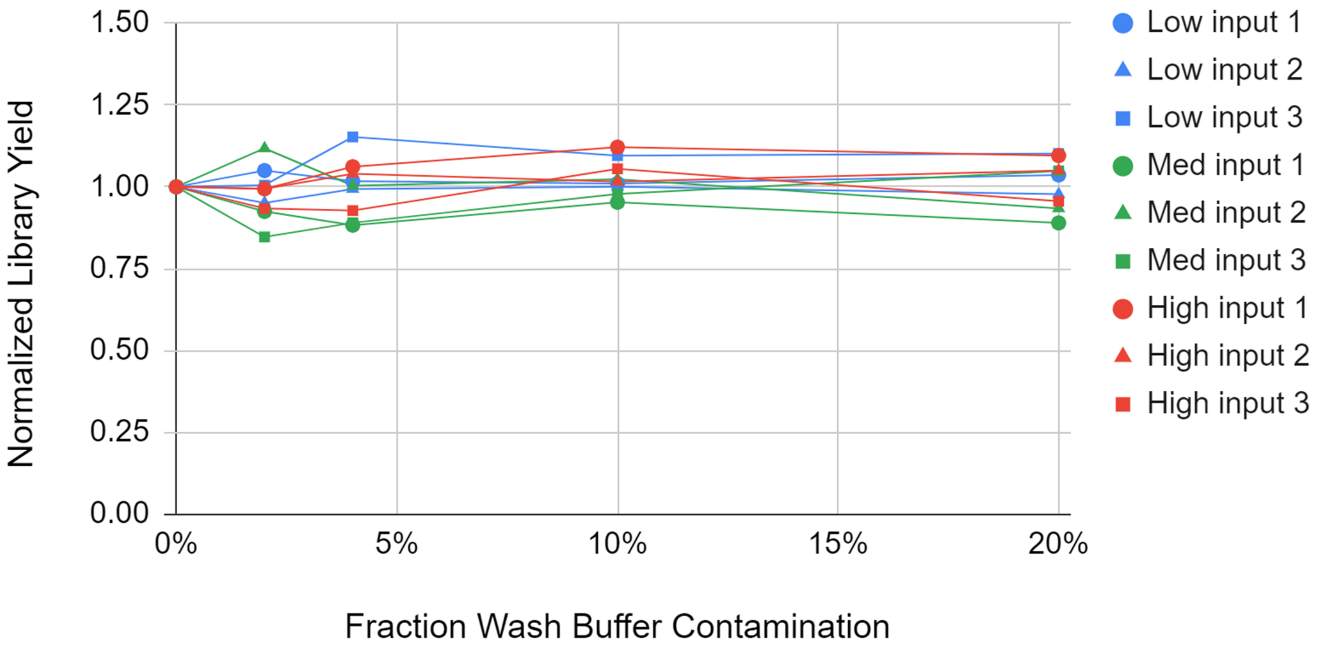

Supplementary Figure 3: Effects of nucleic acid extraction wash buffer contamination on library yield.

Supplementary Table 1: Polynomial coefficients for reagent lots A and B

Coefficient	Reagent lot A	Reagent lot B
B1	−0.6726	0.7324
B2	1.2806	−0.3672
B3	−0.2190	0.2099
B4	0.01249	−0.0198

Supplementary Table 2: ctDNA measurements at various cfDNA input amounts, compared to 15 ng

Cancer specimen	Mean reads at different DNA inputs, PPM				Difference between means		
	Mean (SD) for 2 ng	Mean (SD) for 5 ng	Mean (SD) for 15 ng	Mean (SD) for 30 ng	Between 2 ng and 15 ng (95% CI)	Between 5 ng and 15 ng (95% CI)	Between 30 ng and 15 ng (95% CI)
Breast	51.2 (5.6)*	46.1 (14.3)	38.6 (7.4)	36.6 (2.2)	12.6 (1.98, 23.2)	7.49 (−10.9, 25.9)	−1.95 (−10.75, 6.85)
Colorectal	38.7 (5.3)	31.2 (5.4)	32.3 (3.3)	29.8 (3.7)	6.42 (−0.73, 13.6)	−1.15 (−8.39, 6.08)	−2.52 (−8.19, 3.14)
Melanoma	54.6 (5.7)*	55.8 (5.4)*	45.2 (5.1)	42.8 (2.2)	9.31 (0.61, 18.0)	10.48 (1.99, 19.0)	−2.48 (−8.82, 3.85)
NSCLC	35.9 (13.1)	33.9 (3.3)	33.0 (1.7)	37.3 (0.96)*	2.91 (−12.2, 18.0)	0.98 (−3.28, 5.24)	4.37 (2.09, 6.64)
Renal	50 (13.2)*	35.7 (6.9)	32.6 (0.88)	29.7 (3.7)	17.4 (2.26, 32.5)	3.12 (4.79, 11.0)	−2.87 (7.25, 1.50)

*Denotes the difference in ctDNA signal (PPM) from the 15 ng input is outside of the 95% CI.
